# Dynamic Behaviour of Two-Layered Beam Subjected to Mechanical Load in Thermal Environment

**DOI:** 10.3390/ma18174167

**Published:** 2025-09-05

**Authors:** Simona Doneva, Jerzy Warminski, Emil Manoach

**Affiliations:** 1 Institute of Mechanics, Bulgarian Academy of Sciences, 1113 Sofia, Bulgaria; s.doneva@imbm.bas.bg; 2 Centre of Excellence in Informatics and Information and Communication Technologies, 1113 Sofia, Bulgaria; 3 Department of Applied Mechanics, Lublin University of Technology, 20-618 Lublin, Poland; j.warminski@pollub.pl

**Keywords:** nonlinear dynamics, thermoelastic, reduced model, harmonic balance method, analytical solution, 3 mode reduc-tion, bifurcation diagrams, resonance curves, AUTO

## Abstract

The research investigates a composite beam composed of two layers of different materials and thicknesses subjected to thermal and mechanical loads. Two cases of thermal loading are considered here: uniformly distributed temperature along the whole beam and linearly distributed temperature along the beam thickness. A reduced model of the problem based on the first three beam normal modes is formulated. Additionally, a simplified one-mode reduction model is developed and solved analytically by the harmonic balance method (HBM). A comparison between the results of the three-mode reduction and one-mode reduction models highlights the applicability and limitations of the latter. Differences in the resonance curves produced by these models are thoroughly examined. The correctness of the reduced models is validated through comparison with the created finite element model (FEM) of the bilayer beam. The detailed bifurcation diagrams presented for the three-degrees-of-freedom (3-DOFs) model reveal phenomena such as loss of stability, mode interaction, buckling, and existence of multiple solutions. These findings provide deeper insights into the dynamic behaviour of thin composite beams subjected to mechanical and thermal loads, considering different variations of the temperature distribution.

## 1. Introduction

In recent decades, the dynamic behaviour of thin-walled structures subjected to multiphysics fields has been a topic of increasing investigation. One explanation for this fact is the fast growth of high-technology industries. Typical examples are aerospace industries, chemical and nuclear energy industries, and micro- and nanotechnologies. Many structures applicable in these industries are subjected to the influence of temperature and electrical, magnetic, or fluid fields. The interaction between mechanical fields and concomitant temperature, electrical, or other fields could be significant for the design, proper exploitation, and safety of structures. Often, some structures are built from composite materials consisting of materials with different properties.

Thermomechanical problems are among the most commonly encountered problems in engineering practice and are extensively researched in the scientific literature. The classical books that present the basic problems of thermoelasticity are Boley and Weiner [1] and Nowacki [2].

While early work on thermoelasticity dealt primarily with static problems, the aforementioned industries are accelerating the solution of dynamic thermoelastic problems.

The evolution of dynamic thermoelastic investigations is closely related to the aerospace industry. A series of works studies the phenomena arising due to the combination of airflow, thermal, and mechanical fields acting on aerospace structures. Some of them are systematized in Torton’s book [3].

Other works (referenced as [4,5,6,7,8]) further explore flutter, buckling, and post-buckling behaviours in panels exposed to thermal environments.

A notable characteristic of many works focused on dynamic thermoelastic problems is that the subjects of investigation are often composite structures. Most often, a homogenization procedure is applied for such structures, and the problem is transformed into one of a structure with generalized properties. For example, in [9] and [10], beams with functionally graded materials are considered, and dynamics and stability problems are solved in the case of thermal loading. Also, the dependence of material gradations on the critical buckling temperature is shown.

A large number of works investigate the nonlinear dynamic behaviour of structures subjected to mechanical and thermal loading. The interesting nonlinear phenomena arising in the process of the interaction of these two fields are a challenge to scientists. Such works include references [11,12,13,14,15,16,17,18,19,20,21].

In [11], the equations of motion of a simply supported rectangular plate subjected to thermal and mechanical loads are transformed to three ordinary differential equations by the Galerkin method. The authors have computed numerically various nonlinear features, including Poincaré maps, phase plots, power spectra, and bifurcation diagrams. In [12], thermoelastic vibrations of a rotating microbeam subjected to a laser pulse heat source and sinusoidal heating are considered. Mathematical modelling of the problem is developed using the Euler–Bernoulli beam theory and the generalized theory of thermal conductivity with three relaxation coefficients of time. The closed-form solutions are obtained by Laplace’s transform. Laplace’s transform is again applied in [13] to study the thermoelastic vibration of functionally graded nanobeams interacting with abrupt heat in nonclassical thermoelasticity with phase delays. Systematic and consistent studies of the nonlinear thermoelastic dynamic behaviour of plates are presented in [14,15,16,17,18,19]. In this series of works, the local and global dynamics of homogeneous or composite plates subjected to mechanical and thermal loads are presented. Different plate theories have been used, and coupled and uncoupled problems are considered.

The nonlinear dynamics of the coupled thermoelastic vibrations of isotropic and orthotropic plates are discussed in the work of Yen [20]. Various nonlinear dynamics features, including Poincaré maps, phase diagrams, fractal dimensions, bifurcation diagrams, power spectra, and Lyapunov exponents, are employed to describe the behaviour of these structures.

The effects of the coefficient of thermal expansion (CTE) and the thermal conductivity (TC) on the microbeam’s linear free and forced vibrations are studied in [21]. The authors have used the previously developed scale-dependent linear thermoelastic model of microbeams and have applied the Galerkin method to obtain ordinary differential equations (ODEs). Then, using an analytical approach, the relationships of the bending and temperature vibrations are illustrated, and conclusions about the mechanical energy dissipation and the influence of the CTE and TC on the microbeam’s behaviour are drawn.

Different aspects of dynamic thermoelastic problems of plates and beams are referenced in our previous works devoted to the same topic [22,23,24]. The complete model of thermoelastic vibration of plates considers transversal and longitudinal displacements, taking into account the large displacements, shear deformation, inertia terms due to cross-section rotation, and thermal and mechanical loadings. Based on the described model, the influence of temperature on the first resonance zone and the bifurcation scenario, which results in buckling and chaotic oscillations, is discussed in [22] and [23]. In [24], the reduced model is based on a three-mode reduction and considers the effect of elevated temperature on the plate’s response. On the basis of an analytical model, the buckling phenomenon, post-buckling oscillations, period-doubling bifurcations, and zones of multistable solutions are demonstrated.

Bimaterial beams belong to a special class of composite structures. Usually, the two layers have quite different material properties and thicknesses. Examples of such structures can be found in many micro-electronic devices (MEMSs), energy harvesting devices, and other applications. Most studies of the thermomechanical behaviour of bimaterial beams focus on their static states. Consideration of the bending of bimaterial beams dates back to as early as 1960, as noted in the book by Boley and Weiner [1]. Later, numerous studies emerged regarding the deformation, stresses, and temperature distribution of bi-metallic beams. The static thermoelastic deformation of composite beams is studied in [25,26,27,28]. In [29], the influence of temperature on the vibrations of laminated layers made of two different materials is presented. Essentially, the object of the investigation can be considered as a functionally graded beam.

The authors of this study have applied a model of homogenization of a bilayered beam subjected to temperature changes and periodic dynamic loading [30]. A reduced model, based on a three-mode reduction, was created to study the dynamics of the beam in detail in the frequency domain. The bifurcation analysis showed the possibility of multiple solutions arising and loss of stability. The considered beam was somewhat thick, and obtaining a nonlinear response required large loads. The attempted experimental study confirmed this.

In the present work, the mathematical thermoelastic model was again formulated but for a much thinner beam, which is much more sensitive to thermal and mechanical loads. Therefore, in addition, the case of the temperature linearly distributed along the beam thickness is investigated. This makes the reduced model more complicated but allows the discovery of new phenomena that arise due to temperature influence.

The article is organized as follows: In Section 2, the mathematical model of the dynamic behaviour of a two-layered beam in a thermal environment is presented. The homogenization of the beam is explained, and the basic equations of the beam motion in terms of displacements and rotations are derived. In Section 3, the Galerkin approach is applied, and based on the first three normal modes, a reduced model is created consisting of three nonlinear ordinary differential equations. In Section 4, a particular case of a one-mode reduction model is shown, and the harmonic balance method to solve the obtained nonlinear ordinary differential equation is briefly presented. In Section 5, the FEM is applied for verification of the reduced model. In Section 6, a numerical study based on the continuation method and predictor–corrector steps is performed, and a large number of frequency response curves and bifurcation diagrams are shown. A parametric study of the influence of amplitude and frequency of loading, as well as temperature, on the beam’s response is presented. In Section 7, the applicability of the one-mode reduction model is studied. In Section 8, the main conclusions are drawn.

## 2. Mathematical Model

**Figure 1:**
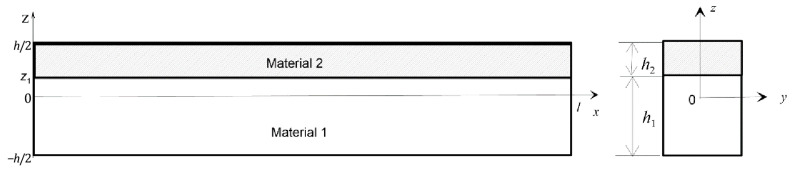
The geometrical scheme of the beam model.

Figure 1 shows a composite beam consisting of two layers of different materials (Material 1 and Material 2) with thicknesses *h*_1_ and *h*_2_. The length of the beam is denoted with *l*, the width with *b*, and the thickness with *h*, where *h* = *h*_1_ + *h*_2_. The coordinate along the z axis of the interface between the layers is denoted with $z_{m}$.

Assuming large displacements and applying the Timoshenko beam theory, the strain and curvature-displacement relationships associated with the mid-axes can be expressed as

(1) $$\varepsilon_{x}^{0}=\frac{\partial u}{\partial x}+\frac{1}{2}{\left(\frac{\partial w}{\partial x}\right)}^{2}\;\varepsilon_{xz}^{0}=\frac{\partial w}{\partial x}-\psi \;\kappa^{0}=-\frac{\partial \psi }{\partial x}$$

and the strain vector is expressed as follows:

(2) $$\varepsilon ={\left\{\varepsilon_{x}^{0}+z\kappa_{x}^{0},\;f(z)\varepsilon_{xz}^{0}\right\}}^{T}$$

where $w\left(x,t\right)$ is the transverse displacement, $\psi \left(x,t\right)$ is the rotation angle, and f(z), according to the Timoshenko beam theory, is a function describing the distribution of the shear strain along the beam thickness. The superscript ^0^ associates the strains $\varepsilon_{x},\;\varepsilon_{xz}$ and the curvature κ with the mid-axes.

### 2.1. Constitutive Equations

The relationships between the stress $S=\left\{\sigma_{x},\sigma_{xz}\right\}$ and strains $\varepsilon =\left\{\varepsilon_{x},\;\varepsilon_{xz}\right\}$, considering the temperature changes, are presented by the following well-known equations (see, for example, the basic book [31] and also the more recent monography [32]):

(3) $$\sigma_{x}^{\left(i\right)}=E^{\left(i\right)}\left[\varepsilon_{x}-\alpha_{T}^{\left(i\right)}(T-T_{i})\right],\;\sigma_{xz}^{\left(i\right)}=G^{\left(i\right)}\varepsilon_{xz}\;i=1,2$$

where *E*^(i)^ is the Young’s modulus of *i*th layer, *G*^(i)^ is the corresponding shear modulus, $\alpha_{T}^{\left(i\right)}$ is the coefficient of thermal expansion, *T* is the current temperature, and $T_{i}$ is the initial (environmental) constant temperature.

### 2.2. Equations of Motion

Starting from the equilibrium equations written for each layer and then following the procedure for homogenization described in [30], the following equations are obtained:

(4) $$\frac{\partial^{2}u}{\partial x^{2}}=G_{u}+G_{u}^{T}$$

(5) $$\frac{\partial^{2}\psi }{\partial x^{2}}-\frac{\bar{Gh}}{\bar{EIZ}}k^{2}\left(\frac{\partial w}{\partial x}-\psi \right)-c_{2}\frac{\partial \psi }{\partial t}-\frac{\bar{\rho I}}{\bar{EIZ}}\frac{\partial^{2}\psi }{\partial t^{2}}=G_{1}^{T}$$

(6) $$k^{2}\frac{\bar{Gh}}{\bar{Eh}}\left(\frac{\partial^{2}w}{\partial x^{2}}-\frac{\partial \psi }{\partial x}\right)-c_{1}\frac{\partial w}{\partial x}-\frac{\bar{\rho h}}{\bar{Eh}}\frac{\partial^{2}w}{\partial t^{2}}=-p_{1}(x,t)+G_{2}^{L}+G_{2}^{T}$$

where *c*_1_ and *c*_2_ denote damping coefficients which are assumed to be proportional to the mass terms in front of the inertia terms. However, the inertia term in the longitudinal direction is neglected.

In Equations (4)–(6), the following notations are introduced:

(7) $$\bar{\mathrm{EI}}=\frac{1}{3}\left\{E^{\left(1\right)}\left(z_{1}^{3}+\frac{h^{3}}{8}\right)+E^{\left(2\right)}\left(\frac{h^{3}}{8}-z_{1}^{3}\right)\right\},$$

(8) $$\bar{E}\bar{I}\bar{Z}=\left[\bar{E}\bar{I}-\frac{{\left(E^{\left(1\right)}-E^{\left(2\right)}\right)}^{2}}{\bar{Eh}}\frac{{\left(h^{\left(1\right)}h^{\left(2\right)}\right)}^{2}}{4}\right]$$

(9) $$G_{u}=-\left(\frac{\partial w}{\partial x}\frac{\partial^{2}w}{\partial x^{2}}\right)-\frac{\left(E^{\left(1\right)}-E^{\left(2\right)}\right)}{\bar{Eh}}\frac{h^{\left(1\right)}h^{\left(2\right)}}{2}\frac{\partial^{2}\psi }{\partial x^{2}}$$

(10) $$G_{u}^{T}=\frac{1}{\bar{Eh}}(E^{\left(1\right)}\alpha_{T}^{\left(1\right)}\frac{\partial \gamma_{T}^{\left(1\right)}}{\partial x}+E^{\left(2\right)}\alpha_{T}^{\left(2\right)}\frac{\partial \gamma_{T}^{\left(2\right)}}{\partial x})$$

(11) $$G_{1}^{T}==-\left(E^{\left(1\right)}\alpha_{T}^{\left(1\right)}\frac{\partial }{\partial x}\chi_{T}^{\left(1\right)}+E^{\left(2\right)}\alpha_{T}^{\left(2\right)}\frac{\partial }{\partial x}\chi_{T}^{\left(2\right)}\right)\frac{1}{\bar{EIZ}}$$

(12) $$G_{2}^{L}=-\left[\left(\frac{\partial u}{\partial x}+0.5{\left(\frac{\partial w}{\partial x}\right)}^{2}\right)+\frac{\left(E^{\left(1\right)}-E^{\left(2\right)}\right)h^{\left(1\right)}h^{\left(2\right)}}{2\bar{Eh}}\frac{\partial \psi }{\partial x}\right]\frac{\partial^{2}w}{\partial x^{2}}$$

(13) $$G_{2}^{T}=\left[E^{\left(1\right)}\alpha_{T}^{\left(1\right)}\gamma_{T}^{\left(1\right)}+E^{\left(2\right)}\alpha_{T}^{\left(2\right)}\gamma_{T}^{\left(2\right)}\right]\frac{\partial^{2}w}{\partial x^{2}}\frac{1}{\bar{Eh}}$$

(14) $$p_{1}(x,t)=\frac{p(x,t)}{\bar{Eh}}$$

(15) $$\begin{array}{l} \gamma_{T}^{\left(1\right)}=\int_{-h/2}^{z_{1}}Tdz,\;\gamma_{T}^{\left(2\right)}=\int_{z_{1}}^{h/2}Tdz;\;\chi_{T}^{\left(1\right)}=\int_{-h/2}^{z_{1}}Tzdz,\;\chi_{T}^{\left(2\right)}=\int_{z_{1}}^{h/2}Tzdz,\\ \gamma_{T}^{\left(1\right)}=\int_{-h/2}^{z_{1}}Tdz,\;\gamma_{T}^{\left(2\right)}=\int_{z_{1}}^{h/2}Tdz\;;\;\chi_{T}^{\left(1\right)}=\int_{-h/2}^{z_{1}}Tzdz,\;\chi_{T}^{\left(2\right)}=\int_{z_{1}}^{h/2}Tzdz, \end{array}$$

The equation describing the temperature propagation in the beam, without considering full coupling with the mechanical field, is

(16) $$\frac{c_{p}^{\left(i\right)}}{\lambda_{T}^{\left(i\right)}}\frac{\partial T}{\partial t}=\frac{\partial^{2}T}{\partial x^{2}}+\frac{\partial^{2}T}{\partial z^{2}}\;\;\;\;\;\;i=1,2$$

where $c_{p}^{\left(i\right)}\;\mathrm{and}\;\lambda_{T}^{\left(i\right)}$ are the heat capacity per unit volume and the thermal conductivity of the *i*th material.

In this work, the temperature field is established, and the temperature is uniformly distributed along the beam length. This means that the temperature does not change in time and the derivatives along the *x* axis are zero. Therefore,

(17) $$G_{u}^{T}=0\;\mathrm{and}\;G_{1}^{T}=0.$$

Concerning the distribution of the temperature along the beam thickness, two cases are considered: when $\Delta T=T-T_{i}$, the temperature is elevated/lowered along the whole beam, and in the second case, the temperature is distributed along the beam thickness following the linear law

(18) $$T(z)=T_{0}+T_{1}z$$

The constants *T*_0_ and *T*_1_ are defined by the temperature boundary conditions.

The following boundary conditions regarding the temperature are defined:

(19) $$\begin{array}{l} T(-h/2)=T_{d}\\ T(h/2)=T_{up} \end{array}$$

where T_d_ and T_up_ denote temperature on the bottom (down) and upper beam surfaces.

In this case, the coefficients in Equation (18) are

(20) $$T_{0}=\frac{T_{up}+T_{d}}{2},\;T_{1}=\frac{T_{up}-T_{d}}{h}$$

Considering this (see Figure 1),

(21) $$\begin{array}{l} h_{2}=h/2-z_{m}\\ h_{1}=z_{m}+h/2 \end{array}$$

it is possible to obtain the case of linear distribution of the temperature along the beam thickness:

(22) $$\begin{array}{l} \gamma_{T}^{\left(1\right)}=\int_{-h/2}^{z_{1}}(T_{0}+T_{1}z)dz=T_{0}h_{1}-0.5T_{1}h_{1}h_{2}\\ \gamma_{T}^{\left(2\right)}=\int_{z_{1}}^{h/2}(T_{0}+T_{1}z)dz=T_{0}h_{2}+0.5T_{1}h_{1}h_{2} \end{array}$$

while the following applies in the case of uniform temperature distribution:

(23) $$\gamma_{T}^{\left(i\right)}=h_{i}\Delta T\;\;\;\;\;\;i=1,2$$

In the further text, the notation Δ*T* means a difference between the beam and ambient temperatures.

## 3. Reduced Model

The mathematical model of the beam vibration is reduced by means of the Galerkin procedure. To transform the partial differential equations into ordinary differential equations, the first three normal modes of the free vibrations of the homogenized beam are used as basis functions.

For convenience, the following dimensionless variables are introduced:

(24) $$\tilde{w}=w/l,\;\tilde{u}=u/l,\;\tilde{\psi }\equiv \psi ,\;\tilde{T}=\left(T-T_{0}\right)/T_{d}\;\;\tilde{x}=x/l,\;\;\;\tilde{t}=tc/l,\;c^{2}=\frac{\bar{EIZ}}{\bar{\rho I}}$$

Then, Equation (4) is transformed into

(25) $$\frac{\partial^{2}\tilde{u}}{\partial {\tilde{x}}^{2}}={\tilde{G}}_{u}$$

(26) $$\frac{\partial^{2}\psi }{\partial x^{2}}-d_{2}\dot{\psi }-\alpha \beta \left(\frac{\partial \tilde{w}}{\partial \tilde{x}}-\psi \right)-\ddot{\psi }=0$$

(27) $$\beta \left(\frac{\partial^{2}\tilde{w}}{\partial x^{2}}-\frac{\partial \psi }{\partial x}\right)-d_{1}\frac{\partial \;\tilde{w}}{\partial \;t}-s\frac{\partial^{2}\tilde{w}}{\partial \;t^{2}}=-{\tilde{p}}_{1}(x,t)+{\tilde{G}}_{2}^{L}+{\tilde{G}}_{2}^{T}$$

where the following notations are introduced in Equations (26) and (27):

(28) $$\beta =k^{2}\frac{\bar{Gh}}{\bar{Eh}},\alpha =\frac{\bar{Eh}l^{2}}{\bar{EIZ}},s=\frac{\bar{\rho h}}{\bar{Eh}}\frac{\bar{EIZ}}{\bar{\rho I}}$$

and Equations (12)–(14) are transformed into

(29) $${\tilde{p}}_{1}(x,t)=\frac{lp(x,t)}{\bar{Eh}},$$

(30) $${\tilde{G}}_{2}^{L}=-\left[\left(\frac{\partial \tilde{u}}{\partial x}+0.5{\left(\frac{\partial \tilde{w}}{\partial x}\right)}^{2}\right)+\frac{\left(E^{\left(1\right)}-E^{\left(2\right)}\right)h^{\left(1\right)}h^{\left(2\right)}}{2\bar{Eh}l}\frac{\partial \psi }{\partial x}\right]\frac{\partial^{2}\tilde{w}}{\partial x^{2}}$$

(31) $${\tilde{G}}_{2}^{T}=h\left[E^{\left(1\right)}\alpha_{T}^{\left(1\right)}\gamma_{T}^{\left(1\right)}+E^{\left(2\right)}\alpha_{T}^{\left(2\right)}\gamma_{T}^{\left(2\right)}\right]\frac{\partial^{2}\tilde{w}}{\partial x^{2}}\frac{1}{\bar{Eh}}$$

where *d*_1_ and *d*_2_ are damping coefficients. For simplicity, in the text below “tilde” is omitted. The solution of Equations (26) and (27) is sought in the following form:

(32) $$w(x,t)=\sum_{i=1}^{3}W_{i}(x)q_{i}(t),\;\psi (x,t)=\sum_{i=1}^{3}\Psi_{i}(x)q_{i}(t)$$

where *W*_i_(*x*) and *Ψ*_i_(x) are the normal modes of the beam vibrations—the solution of Equations (26) and (27) with a zero right-hand side and satisfying the boundary conditions. Substituting Equation (32) into Equations (26) and (27), using the orthogonality condition, the following system of nonlinear ordinary differential equations is obtained:

(33) $${\ddot{q}}_{n}+2\xi_{n}{\dot{q}}_{n}+\omega_{n}^{2}q_{n}=F_{n}^{p}+F_{n}^{L}+F_{n}^{T}$$

In Equation (33), subscript “n” indicates the mode number, *ω*_n_ represents the natural frequencies of the linear elastic undamped bimaterial Timoshenko beam, *ξ*n represents the modal damping coefficients, and

(34) $$F_{n}^{p}=-\int_{0}^{1}W_{n}(x)p_{1}(x,t)dx,\;$$

(35) $$F_{n}^{L}=\int_{0}^{1}\left[W_{n}(x)G_{2}^{L}(x,t)\right]dx\;$$

(36) $$F_{n}^{T}=\int_{0}^{1}\left[W_{n}(x)G_{2}^{T}(x,t)\right]dx$$

Following the procedure developed in [30], it is found that

(37) $$G_{2}^{L}=-\int_{0}^{1}{\left(\frac{\partial w}{\partial \xi }\right)}^{2}d\xi \frac{\partial^{2}w}{\partial x^{2}}$$

This denotes

(38) $$I_{ij}=\int_{0}^{1}\frac{\partial W_{i}}{\partial x}\frac{\partial W_{j}}{\partial x}dx$$

then, substituting Equation (32) into Equation (37), the following expression for the nonlinear force vector $G_{2}^{L}$ is determined:

(39) $$\begin{array}{l} G_{2}^{L}=-0.5(I_{11}{w_{1}}^{\prime \prime }q_{1}^{3}+I_{22}{w_{2}}^{\prime \prime }q_{2}^{3}+I_{33}{w_{3}}^{\prime \prime }q_{3}^{3})-\\ -0.5\left[\left(I_{11}w_{2}^{\prime \prime }+2I_{12}w_{1}^{\prime \prime }\right)q_{1}^{2}q_{2}+(I_{22}w_{1}^{\prime \prime }+2I_{12}w_{2}^{\prime \prime })q_{1}q_{2}^{2}+(I_{33}w_{1}^{\prime \prime }+2I_{13}w_{3}^{\prime \prime })q_{1}q_{3}^{2}\right]\\ -0.5\left[\left(I_{11}w_{3}^{\prime \prime }+2I_{13}w_{1}^{\prime \prime })q_{1}^{2}q_{3}+(I_{22}w_{3}^{\prime \prime }+2I_{23}w^{\prime \prime })q_{2}^{2}q_{3}+(I_{33}w_{2}^{\prime \prime }q_{2}q_{3}^{2}+2I_{23}w_{3}^{\prime \prime }\right)\right]\\ -0.5(2I_{23}w_{1}^{\prime \prime }+2I_{12}w_{3}^{\prime \prime }+2I_{13}w_{2}^{\prime \prime })q_{1}q_{2}q_{3} \end{array}$$

For term $G_{2}^{T}$, which accounts for the influence of temperature, the case of linear temperature distribution along the beam thickness is considered. When *T*_up_ = *T*_d_, obviously, *T*_1_ = 0, and the beam is just subjected to elevated/lowered temperature Δ*T*.

Then

(40) $$G_{2}^{T}=\left[E^{\left(1\right)}\alpha_{T}^{\left(1\right)}\gamma_{T}^{\left(1\right)}+E^{\left(2\right)}\alpha_{T}^{\left(2\right)}\gamma_{T}^{\left(2\right)}\right]\frac{\partial^{2}w}{\partial x^{2}}\frac{1}{\bar{Eh}}$$

can be presented in the form

(41) $$G_{2}^{T}=E_{T}\frac{\partial^{2}w}{\partial x^{2}}\frac{1}{\bar{Eh}}$$

where

(42) $$E_{T}=\left[E^{\left(1\right)}\alpha_{T}^{\left(1\right)}\gamma_{T}^{\left(1\right)}+E^{\left(2\right)}\alpha_{T}^{\left(2\right)}\gamma_{T}^{\left(2\right)}\right]\frac{1}{\bar{Eh}}=E_{T1}T_{0}+E_{T2}T_{1}$$

(43) $$E_{T1}=\frac{E^{\left(1\right)}h_{1}\alpha_{T}^{\left(1\right)}+E^{\left(2\right)}h_{2}\alpha_{T}^{\left(2\right)}}{\bar{Eh}},\;E_{T2}=\frac{h_{1}h_{2}}{h}\frac{E^{\left(2\right)}\alpha_{T}^{\left(2\right)}-E^{\left(1\right)}\alpha_{T}^{\left(1\right)}}{\bar{Eh}}$$

Therefore

(44) $$\begin{array}{l} F_{n}^{T}=\int_{0}^{1}\left[W_{n}(x)G_{2}^{T}(x,t)\right]dx=E_{T}\left(\int_{0}^{1}W_{n}({W_{1}}^{\prime \prime }q_{1}+{W_{2}}^{\prime \prime }q_{2}+{W_{3}}^{\prime \prime }q_{3})dx\right)=\\ (E_{T})\times ({R_{n}}_{1}q_{1}+R_{n2}q_{2}+{R_{n}}_{3}q_{3})\;n=1,2,3 \end{array}$$

(45) $$R_{ij}=\left(\int_{0}^{1}W_{i}({W_{j}}^{\prime \prime })dx\right)\;i,j=1,2,3$$

Introducing the notations

(46) $$\begin{array}{l} D_{1n1}=E_{T1}\int_{0}^{1}W_{n}{W_{1}}^{\prime \prime }dx,\;D_{1n2}=E_{T1}\int_{0}^{1}W_{n}{W_{2}}^{\prime \prime }dx,\;D_{1n3}=E_{T1}\int_{0}^{1}W_{n}{W_{3}}^{\prime \prime }dx\\ D_{2n1}=E_{T2}\int_{0}^{1}W_{n}{W_{1}}^{\prime \prime }dx,\;D_{2n2}=E_{T2}\int_{0}^{1}W_{n}{W_{2}}^{\prime \prime }dx,\;D_{2n3}=E_{T2}\int_{0}^{1}W_{n}{W_{3}}^{\prime \prime }dx \end{array}$$

and taking into account Equations (34)–(46), the set of ODEs representing the three-mode reduced model of the large-amplitude thermoelastic vibrations of the bimaterial beam takes the following final form:

(47) $$\begin{array}{l} {\ddot{q}}_{n}+2\xi_{n}{\dot{q}}_{n}+\omega_{n}^{2}q_{n}+C_{(n)111}q_{1}^{3}+C_{(n)222}q_{2}^{3}+C_{(n)333}q_{3}^{3}+C_{(n)112}q_{1}^{2}q_{2}+C_{(n)113}q_{1}^{2}q_{3}+\\ C_{(n)122}q_{1}q_{2}^{2}+C_{(n)223}q_{2}^{2}q_{3}+C_{(n)133}q_{1}q_{3}^{2}+C_{(n)233}q_{2}q_{3}^{2}+C_{(n)123}q_{1}q_{2}q_{3}-\;n=1,2,3\\ \left(T_{0}D_{1n1}+T_{1}D_{2n1}\right)q_{1}+\left(T_{0}D_{1n2}+T_{1}D_{212}\right)q_{2}+\left(T_{0}D_{1n3}+T_{1}D_{2n3}\right)q_{3}=-F_{n}\sin(\omega t)\;\;\; \end{array}$$

where

(48) $$\begin{array}{l} C_{(n)ijj}=0.5\int_{0}^{1}W_{n}(I_{22}{W_{i}}^{\prime \prime }+2I_{ij}W_{j}^{\prime \prime })dx,\;C_{(n)123}=\int_{0}^{1}W_{n}(I_{23}{W_{1}}^{\prime \prime }+I_{12}{W_{3}}^{\prime \prime }+I_{13}{W_{2}}^{\prime \prime })dx\\ D_{1ni}=E_{T1}\int_{0}^{1}W_{n}{W_{i}}^{\prime \prime }dx,\;D_{2ni}=E_{T2}\int_{0}^{1}W_{n}{W_{i}}^{\prime \prime }dx\;i=1,2,3;\;n=1,2,3 \end{array}$$

The values of the variables, involved in Equation (47) for the cases considered in the numerical examples are given in Appendix A.

## 4. Analytical Solutions for the One-Mode Reduction Model

In parallel with the 3-DOFs model, a one-mode reduction model is created and studied separately. The goal of this analysis is to derive an analytical solution to the equation of motion and compare the results with those based on three-mode reduction. The analytical solution allows us to obtain in a closed form the dependence of different parameters of the problem—excitation frequency, amplitude of the load, and temperature—on the system response. This is an essential advantage of such a reduction.

The 1-DOF model is obtained automatically by substituting in Equation (47) q_2_ = 0 and q_3_ = 0 and just taking into account the generalized coordinate q_1_.

In this case, Equation (47) for *n* = 1 is transformed into the following equation:

(49) $${\ddot{q}}_{1}+2\xi_{1}{\dot{q}}_{n}+\omega_{1}^{2}q_{1}+C_{(1)111}q_{1}^{3}-\left(T_{0}D_{111}+T_{1}D_{211}\right)q_{1}=-F_{1}\sin(\omega t)\;\;\;$$

The reduced nonlinear one-degree-of-freedom model with cubic nonlinearity is analytically solved using the harmonic balance method [33]. The solution to the equation is sought in the following form:

(50) $$x=A_{1}\left(t\right)\sin\omega t+A_{2}\left(t\right)\cos\omega t$$

where *A_1_* and *A_2_* are unknown amplitudes assumed as slow functions of time.

Substituting Equation (50) into Equation (49), neglecting higher-order harmonics and assuming that the terms ${\ddot{A}}_{1}$ and ${\ddot{A}}_{2}$, along with terms involving amplitude derivatives of an order higher than one, are small quantities of a higher order, a set of first-order equations is obtained:

(51) $$\begin{array}{l} (\omega_{1}^{2}-\omega^{2})A_{1}-2\xi \omega_{1}\omega A_{2}+\frac{3}{4}\gamma (A_{1}^{3}+A_{1}A_{2}^{2})+\lambda \Delta TA_{1}-2\omega {\dot{A}}_{2}+2\xi \omega_{1}{\dot{A}}_{1}=P\\ (\omega_{1}^{2}-\omega^{2})A_{2}+2\xi \omega_{1}\omega A_{1}+\frac{3}{4}\gamma (A_{2}^{3}+A_{1}^{2}A_{2})+\lambda \Delta TA_{2}+2\omega {\dot{A}}_{1}+2\xi \omega_{1}{\dot{A}}_{2}=0 \end{array}$$

Equation (51) comprises first-order time derivatives of the amplitudes and can be transformed into the so-called modulation equations, as they represent modulation of the amplitudes A_1_ and A_2_:

(52) $$\begin{array}{l} {\dot{A}}_{1}\left(t\right)=-\frac{1}{16\left(\xi^{2}{\omega_{1}}^{2}+\omega^{2}\right)}\left(\begin{array}{l} -8P\xi \omega_{1}+8\lambda \Delta T\xi \omega_{1}A_{1}\left(t\right)+8\xi \omega_{1}^{3}A_{1}\left(t\right)+8\xi \omega_{1}\omega^{2}A_{1}\left(t\right)+\\ 6\gamma \xi \omega_{1}A_{1}^{3}\left(t\right)+8\lambda \Delta T\omega A_{2}\left(t\right)+8\omega_{1}^{2}\omega A_{2}\left(t\right)-16\xi^{2}\omega_{1}^{2}\omega A_{2}\left(t\right)-\\ 8\omega^{3}A_{2}\left(t\right)+6\gamma \omega A_{1}^{2}\left(t\right)A_{2}\left(t\right)+6\gamma \xi \omega_{1}A_{1}\left(t\right)A_{2}^{2}\left(t\right)+6\gamma \omega A_{2}^{3}\left(t\right) \end{array}\right)\\ {\dot{A}}_{2}\left(t\right)=-\frac{1}{16\left(\xi^{2}{\omega_{1}}^{2}+\omega^{2}\right)}\left(\begin{array}{l} 8P\omega -8\lambda \Delta T\omega A_{1}\left(t\right)-8\omega_{1}^{2}\omega A_{1}\left(t\right)+16\xi^{2}\omega_{1}^{2}\omega A_{1}\left(t\right)+8\omega^{3}A_{1}\left(t\right)-\\ 6\gamma \omega A_{1}^{3}\left(t\right)+8\lambda \Delta T\xi \omega_{1}A_{2}\left(t\right)+8\xi \omega_{1}^{3}A_{2}\left(t\right)+8\xi \omega_{1}\omega^{2}A_{2}\left(t\right)+\\ 6\gamma \xi \omega_{1}A_{1}^{2}\left(t\right)A_{2}\left(t\right)-6\gamma \omega A_{1}\left(t\right)A_{2}^{2}\left(t\right)+6\gamma \xi \omega_{1}A_{2}^{3}\left(t\right) \end{array}\right) \end{array}$$

where $\lambda =\left(T_{0}D_{111}+T_{1}D_{211}\right)$, and $\gamma =C_{(1)111}$.

For a steady state, derivatives of amplitude are zero and, therefore, after some analytical computations, a cubic algebraic equation for the amplitude modulus $A=\sqrt{{A_{1}}^{2}+{A_{2}}^{2}}$ is obtained:

(53) $$\begin{array}{l} -16{F_{1}}^{2}+9z^{3}\gamma^{2}+z^{2}\left(24\gamma \lambda \Delta T+24\gamma \omega_{1}^{2}-24\gamma \omega^{2}\right)+\\ z\left(16\lambda^{2}\Delta T^{2}+32\lambda \Delta T\omega_{1}^{2}+16\omega_{1}^{2}-32\lambda \Delta T\omega^{2}-32\omega_{1}^{2}\omega^{2}+64\xi^{2}\omega_{1}^{2}\omega^{2}+16\omega^{4}\right)=0 \end{array}$$

where *z* = *A*^2^.

The modulation Equation (52) can be written in a short form as the following:

(54) $$\begin{array}{l} \frac{dA_{1}}{dt}=f_{1}\left(A_{1},A_{2}\right)\\ \frac{dA_{2}}{dt}=f_{2}\left(A_{1},A_{2}\right) \end{array}$$

Stability analysis is based on the Jacobian matrix and involves evaluating how a system responds to small disturbances near an equilibrium (fixed) point. The type and stability of each singular point depends on the eigenvalues (roots) of the Jacobian matrix:

(55) $$J=\left(\begin{array}{cc} \frac{\partial f_{1}}{\partial A_{1}} & \frac{\partial f_{1}}{\partial A_{2}}\\ \frac{\partial f_{2}}{\partial A_{1}}\; & \frac{\partial f_{2}}{\partial A_{2}} \end{array}\right)$$

Eigenvalues may be real or complex numbers, leading to different dynamic behaviours. The solution is unstable if at least one real part of the roots is positive.

The details of the stability analysis are omitted here for the sake of simplicity.

This algebraic equation connects the excitation amplitude and frequency as well as the temperature loading and allows the resonance curves to be obtained and other parametric studies of the bimaterial beam to be performed. The analytical solution obtained by the HBM will be compared with the solution for the more advanced three-mode model obtained directly from ODEs by the continuation method [34]. This comparison gives information about the applicability of one-mode reduction and demonstrates the influence of the higher-order modes on the beam dynamics, stability of the solutions, and temperature influence.

## 5. Finite Element Model Validation

The developed model for the dynamic behaviour of the thermoelastic bimaterial beam is validated through finite element modelling. A three-dimensional model of the bimaterial beam is created using the commercial software ANSYS 2021/R2 (Canonsburg, PA, USA) [35]. The three-dimensional finite element SOLID 226 is used for discretization of the beam. It is applicable to a lot of coupled-fields analyses including Structural–Thermal. This element has twenty nodes with up to six degrees of freedom per node, which allows computations with a high accuracy.

First, a mesh convergence study focusing on the natural frequencies, critical buckling temperature, and maximum displacement is performed. Three different levels of mesh density are created: coarse mesh (6000 elements and 8844 nodes), medium mesh (32,000 elements and 157,889 nodes), and fine mesh (256,000 elements and 295,569 nodes). The results obtained by these three different models show that the quantities of interest converge with mesh refinement, with relative changes being much less than 1% between the medium and fine meshes—see Table 1.

**Figure 2:**
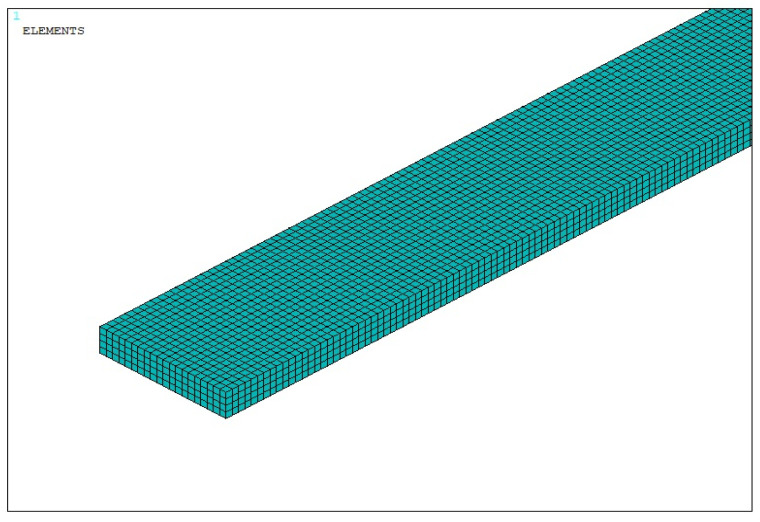
Discretization of the beam model.

Based on the obtained results, we verified that the medium-density mesh used in the main simulations provides a balance between accuracy and computational cost and is fine enough to capture the response of the structure. The finite element discretization of the beam is illustrated in Figure 2.

The analysed beam is composed of two layers: the bottom layer is made of aluminium alloy Al 1050 (Material 1), and the upper layer is made of copper C 12,500 (Material 2). The dimensions of the beam shown in Figure 1 are as follows:

*l* = 0.4 m, *h* = 0.004 m, *h*_1_ = 0.003 m, *h*_2_ = 0.001 m, *b* = 0.02 m

with the following material parameters of each layer:

*E*^(1)^ = 7.0 × 10^10^ N/m^2^, ρ^(1)^ = 2778 kg/m^3^, ν_1_ = 0.34, α_T_^(1)^ = 23.9 × 10^−6^ 1/K

E^(2)^ = 12.8 × 10^10^ N/m^2^, ρ^(2)^ = 8940 kg/m^3^, ν_2_ = 0.34, α_T_^(2)^ = 16.7 × 10^−6^ 1/K

To express the nonlinear phenomena more clearly, clamped–clamped boundary conditions are assumed in the analysis.

The thickness of the beam is discretized into four layers: one layer for copper and three layers for aluminium.

The initial task is to perform a modal analysis to determine the natural frequencies and the corresponding mode shapes. The natural frequencies calculated by the finite element model are compared with those obtained analytically for the reduced model, and the results are presented in Table 2.

A difference of about 7% was also recorded in a previous paper using homogenization of the beam and the reduced model [30].

The second task is to compare the forced response of the beam subjected to harmonic and thermal loads.

The Finite Element Method (FEM) is a powerful tool for analysing complex structures and solving nonlinear differential equations, but it requires significant computational resources and time to obtain accurate results. The limitations in terms of computational power and time can restrict the number of cases that can be studied using the FEM. However, the primary objective of FEM computations is to highlight the main trends of the analysed processes and validate the mathematical models used.

**Figure 3:**
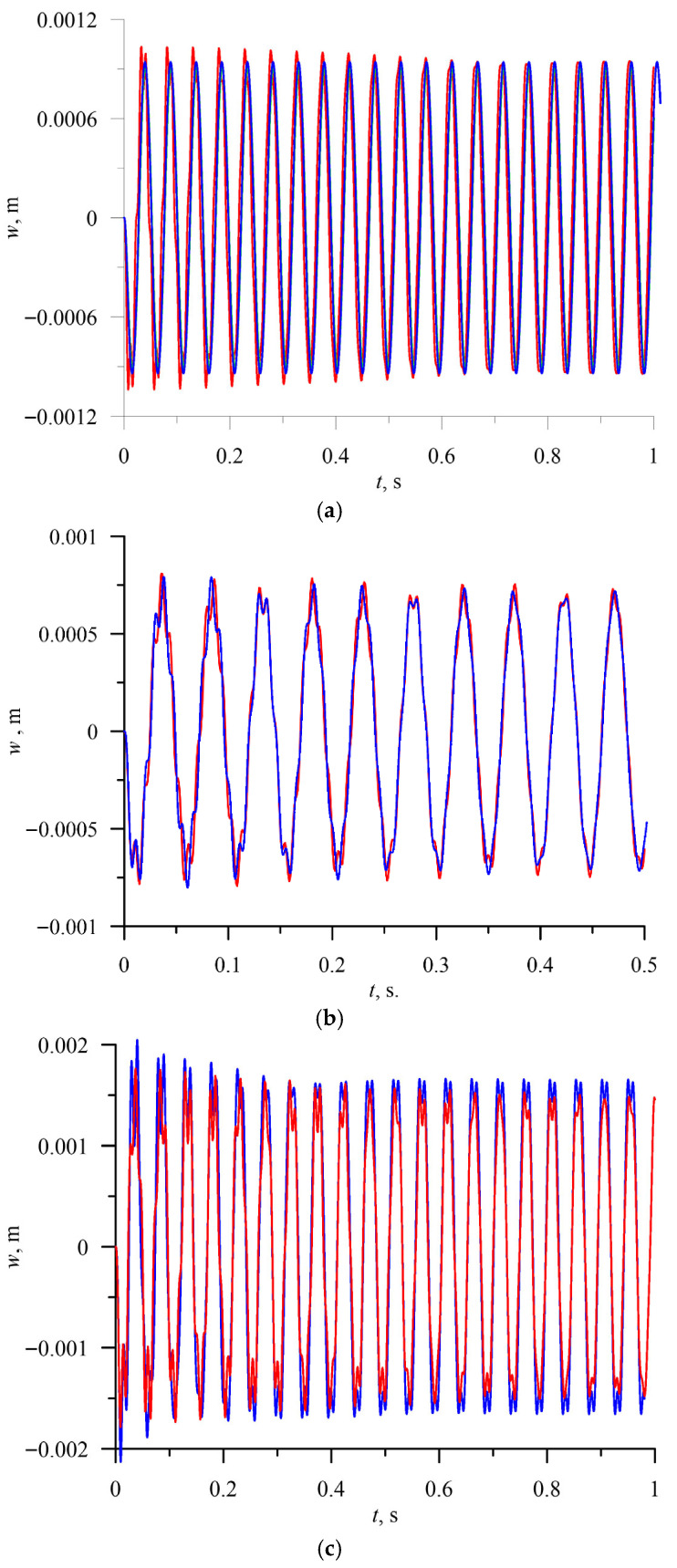
(**a**) Time history diagram of the response of the beam centre—red colour, ANSYS; blue colour, reduced model (3-mode reduction). T = 5, P = 5000 N/m^2^, and ω = 130 rad/s. (**b**) Time history diagram of the response of the beam centre—red colour, ANSYS; blue colour, reduced model (3-mode reduction). T = 10, P = 500 N/m^2^, and ω = 130 rad/s. (**c**) Time history diagram of the response of the bimaterial beam subjected to harmonic loading. P = 5000, ω = 130 rad/s, and *T* = 10. Comparison between the reduced model and finite element model. Blue colour—reduced model; red colour—ANSYS.

The comparison of the time history diagrams for the bimaterial beam subjected to harmonic loads with different amplitudes and temperatures, obtained using the reduced model of the beam and the finite element model, is presented in Figure 3a–c.

The presented modal analysis and computed dynamic time responses for the finite element model and the reduced three-mode model are in good agreement. The observed deviation results from different assumptions in both models. However, the differences are acceptable, and the correctness of the theoretical results is confirmed. A more advanced bifurcation analysis and study of several interesting phenomena will be performed based on the reduced models.

## 6. Nonlinear Dynamics Based on the Three-Mode Reduction Model

**Figure 4:**
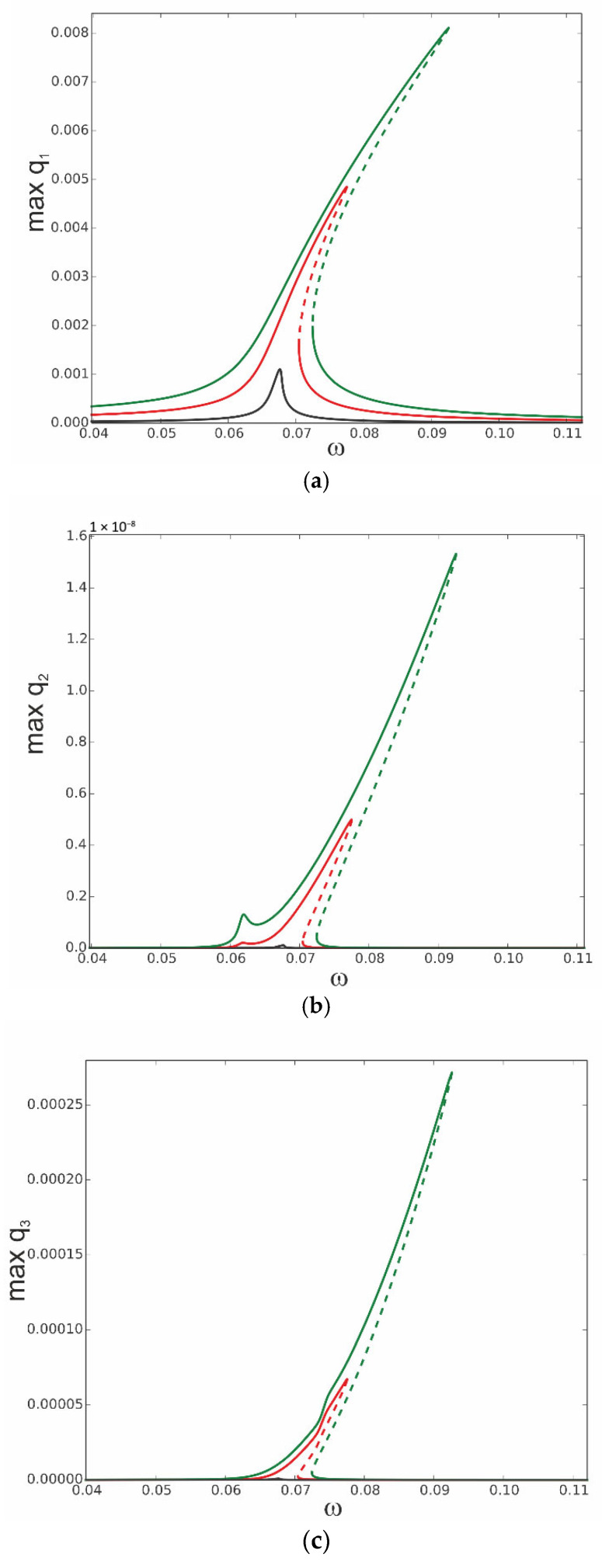
Resonance curves around the first natural frequency for large mechanical loading: f_1_ = 0.1 × 10^−6^—black; f_1_ = 0.5 × 10^−6^—red; f_1_ = 0.1 × 10^−5^—green; ∆*T* = 0; (**a**) coordinate q_1_, (**b**) coordinate q_2_, and (**c**) coordinate q_3_.

The equations of motion (31) of the reduced model of the thermoelastic beam’s dynamics are solved numerically, and the continuation technique with predictor–corrector steps is used to obtain bifurcation curves [34]. In this case, the stability of the periodic solutions is determined by Floquet multipliers computed for the three ordinary differential equations of motion. The analysis starts with computation of the resonance curves, neglecting influence of the thermal loading, assuming *T*_up_ = 0 and *T*_d_ = 0. The curves are plotted for the case when the first mode is excited directly; thus, periodic force occurs on the right side of the first equation F_1_ = f_1_sinωt, while F_2_ = 0 and F_3_ = 0. This is the most important case because the first mode plays a crucial role in the dynamics. As the system is nonlinear, the other modes are coupled, and therefore, they are indirectly activated as well. The three levels of excitation are selected to observe the beam’s response for the first q1 (Figure 4a), second q2 (Figure 4b), and third q3 (Figure 4c) generalized coordinates. For the very small level of excitation amplitude (black curve), we observe almost a linear resonance curve for q1 with negligible involvement of q2 and q3 coordinates.

As excitation increases, the curves corresponding to q_1_ bend towards higher frequencies (red and green colour) and coordinates q_2_ and q_3_ are much more involved, however, with much larger participation of coordinate q3 than q2 (note that in Figure 4b, the scale is of 1 × 10^−8^ order).

Thus, considering that for moderate vibrations the modal interactions are rather negligible, the results based on the one-mode reduction model can accurately capture the real system response, as presented in Section 4. Then, the solution can be determined analytically with very good accuracy.

**Figure 5:**
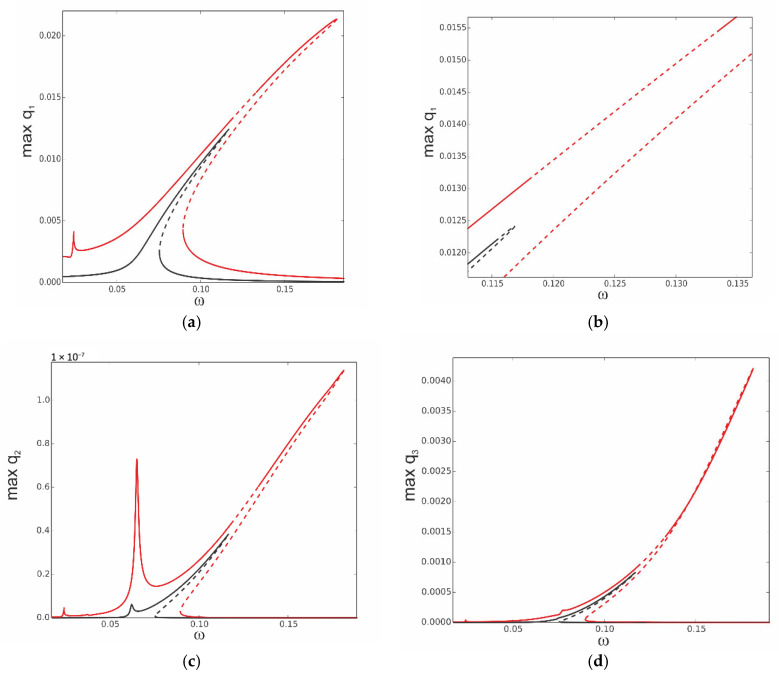
(**a**,**b**) Resonance curves around the first natural frequency for large mechanical loading: f_1_ = 0.2 × 10^−5^—black; f_1_ = 0.1 × 10^−4^—red; ∆*T* = 0; (**a**) coordinate q_1_ and (**b**) zoomed-in view of coordinate q_1_ in vicinity of unstable zone. (**c**,**d**) Resonance curves around the first natural frequency for large mechanical loading: f_1_ = 0.2 × 10^−5^—black; f_1_ = 0.1 × 10^−4^—red; ∆*T* = 0; (**c**) coordinate q_2_ and (**d**) coordinate q_3_.

More complicated dynamics occur for large values of excitation, as presented in Figure 5. The instability of the resonance curves occurs for larger oscillation amplitudes, located close to the peak of the black resonance curve (f_1_ = 0.2 × 10^−5^) and in the middle of the red curve (f_1_ = 0.1 × 10^−4^), as presented in detail in the zoomed-in view in Figure 5b. Consequently, the instability is present for coordinate q_2_ in Figure 5c and coordinate q_3_ in Figure 5d.

**Figure 6:**
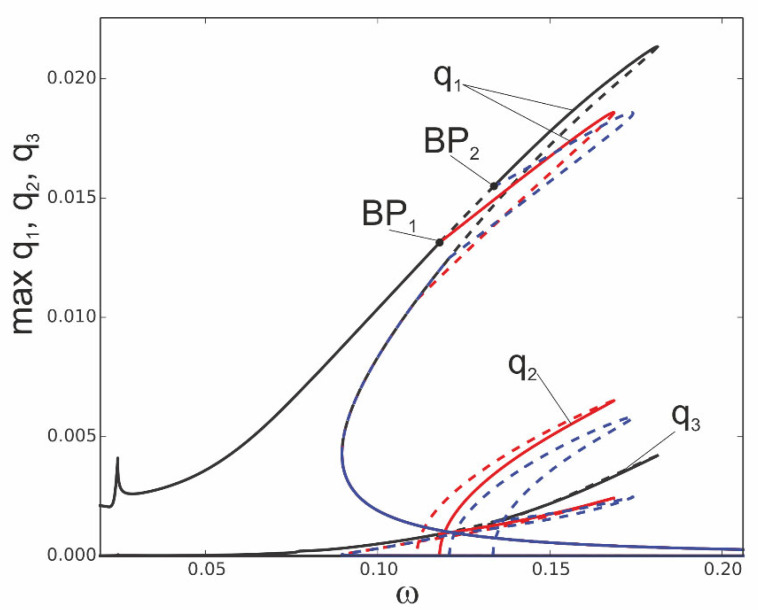
Resonance curves around the first natural frequency for large mechanical loading: f_1_ = 0.1 × 10^−4^, f_2_ = 0, f_3_ = 0, and ∆*T* = 0; coordinates q_1_, q_2_, and q_3_ with different branches indicated by black, red, and blue colours and bifurcation points BP_1_ and BP_2_.

The following question arises: what happens to the system if it works in the instability zone? In Figure 6, the solutions are determined around the frequency corresponding to bifurcation points BP_1_ and BP_2_. Starting from the bifurcation point BP_1_, an additional stable solution arises (red line), and this is an effect of a strong involvement of coordinate q_2_. From the bifurcation point BP2, only unstable solutions arise, plotted by the blue dashed line. This means that for large vibrations, bifurcation points on the resonance curve occur, and from them, new solutions arise. The second vibration mode q_2_ is strongly activated. Out of this zone, the first coordinate q_1_ interacts mainly with the third mode q_3_, while the second mode remains at a very small level. The phenomenon of stability loss can be observed for the model with three degrees of freedom, and it is not observed for the one-mode reduction (Section 4).

**Figure 7:**
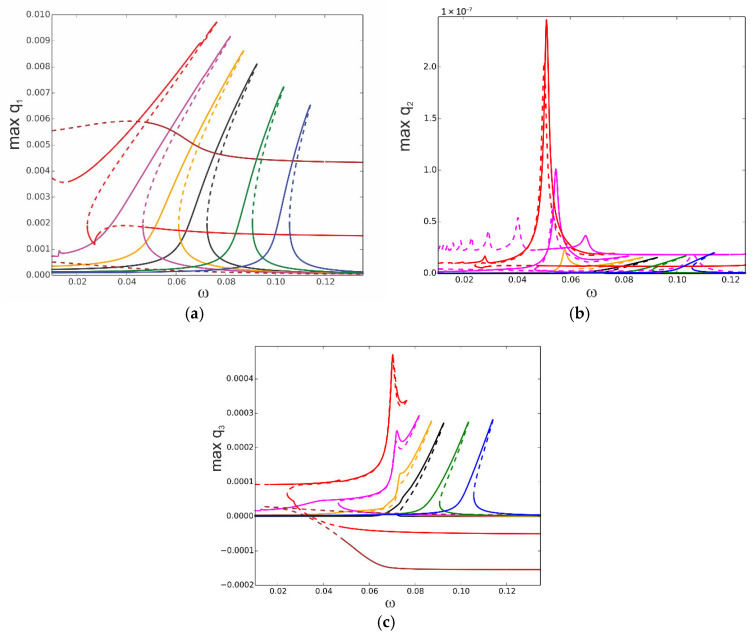
Resonance curves around the first natural frequency for mechanical loading f_1_ = 0.1× 10^−5^ and different values of elevated temperature: Δ*T* = 0—black; Δ*T* = 5—orange; Δ*T* = 10—pink; Δ*T* = 15—red; Δ*T* = 20—brown; Δ*T* = −10—green; Δ*T* = −20—blue; (**a**) coordinate q_1_, (**b**) coordinate q_2_, and (**c**) coordinate q_3_.

The temperature influence in the vicinity of the first natural frequency is at first demonstrated for temperature uniformly distributed through the beam volume and for f_1_ = 0.1 × 10^−5^, i.e., for relatively large vibrations but without the stability loss discussed above. In Figure 7, the black curve represents the reference resonance curve for Δ*T* = 0. The “hot” colour curves correspond to elevated temperatures Δ*T* = 5 (orange), Δ*T* = 10 (pink), Δ*T* = 15 (red), and Δ*T* = 20 (brown), and “cold” colours correspond to decreased temperatures Δ*T* = −10 (green) and Δ*T* = −20 (blue). A decreased temperature below the reference Δ*T* = 0 leads to a shift of the resonance curves towards higher frequencies and to amplitude reduction. An elevated temperature up to Δ*T* = 15 increases the amplitudes and shifts the resonance curves towards lower frequencies. At Δ*T* = 15, the beam buckles, and a new branch arises from the bifurcation point. A further increase in the temperature up to Δ*T* = 20 “destroys” the classical resonance curve, and just post-buckling oscillations exist (brown colour) with the partially stable branch and with the unstable part for lower frequencies. The resonance curves are also shifted correspondingly for q_2_ (Figure 7b) and q_3_ (Figure 7c). Their involvement in the response is more evident when the temperature is increased.

**Figure 8:**
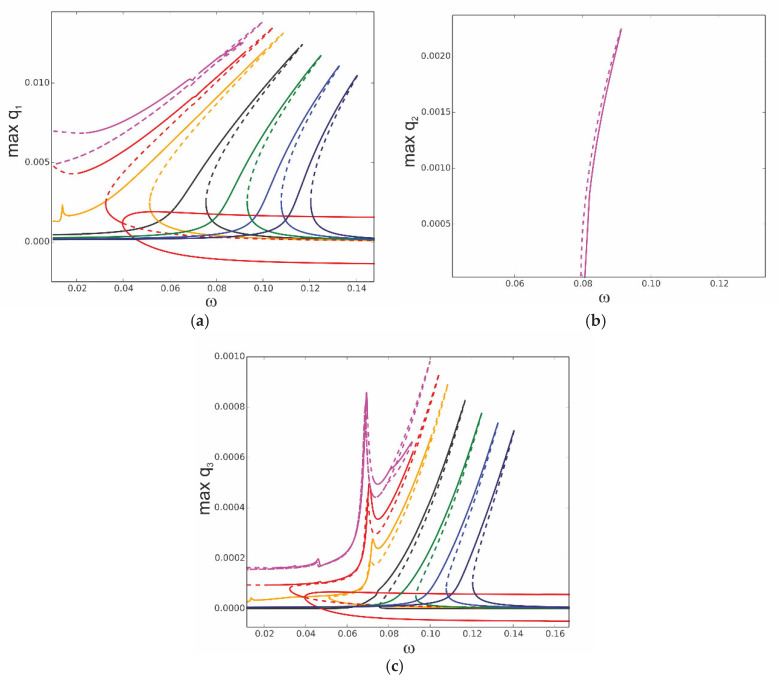
Resonance curves around the first natural frequency for mechanical loading f_1_ = 0.2 × 10^−5^ and different values of elevated temperature: Δ*T* = 0—black; Δ*T* =10—orange; Δ*T* =15—red; Δ*T* =20—magenta; Δ*T* =−10—green; Δ*T* =−20—blue; Δ*T* =−30—navy blue; (**a**) coordinate q_1_, (**b**) coordinate q_2_, and (**c**) coordinate q_3_.

The influence of the temperature is also tested for twice larger mechanical loading for f_1_ = 0.2 × 10^−5^. The resonance curves are plotted for selected values of temperatures, again in “hot” colours for positive and “cold” colours for negative temperatures (Figure 8). For temperatures ∆*T* = −10, ∆*T* = −20, and ∆*T* = −30, the resonance curves are shifted towards higher frequencies, presenting hardening and smaller values of amplitudes (∆*T* =−10—green; ∆*T* = −20—blue; ∆*T* = −30—navy blue). In the case of elevated temperature, the response becomes more complex. For ∆*T* = 10, the curve is shifted towards lower frequencies (orange curve) with an additional secondary resonance demonstrated by a peak in the resonance curve. A further temperature increase up to ∆*T* = 15 changes the curve essentially (red colour). First of all, two instability zones on the upper branch of the curve arise, at the beginning of the curve and close to the peak. The additional phenomenon is a bifurcation of the solution with two stable and one unstable branches, which represents buckling of the beam in a positive or negative direction. The solutions for the post-buckled state are fully stable in contrast to that presented in Figure 7a for ∆*T* = 15 where the post-buckling instability zone exists.

For ∆*T* = 20, the classical shape of the resonance curve is “destroyed” (magenta curve), and close to the top of the upper branch, an additional stable solution arises. This occurs due to the strong interaction with the second mode, as presented in Figure 8b. Values of q_2_ corresponding to other resonance curves are very small, and therefore, they are invisible in Figure 8b. The involvement of the third mode is presented in Figure 8c, and it remains in the same order for all discussed cases.

Analysing the above results, presented in Figure 7 and Figure 8, we may conclude that the resonance characteristics are changed qualitatively if the temperature is elevated above about Δ*T* = 15. To check how the response changes while the temperature is varied, the bifurcation diagrams against Δ*T* are computed.

**Figure 9:**
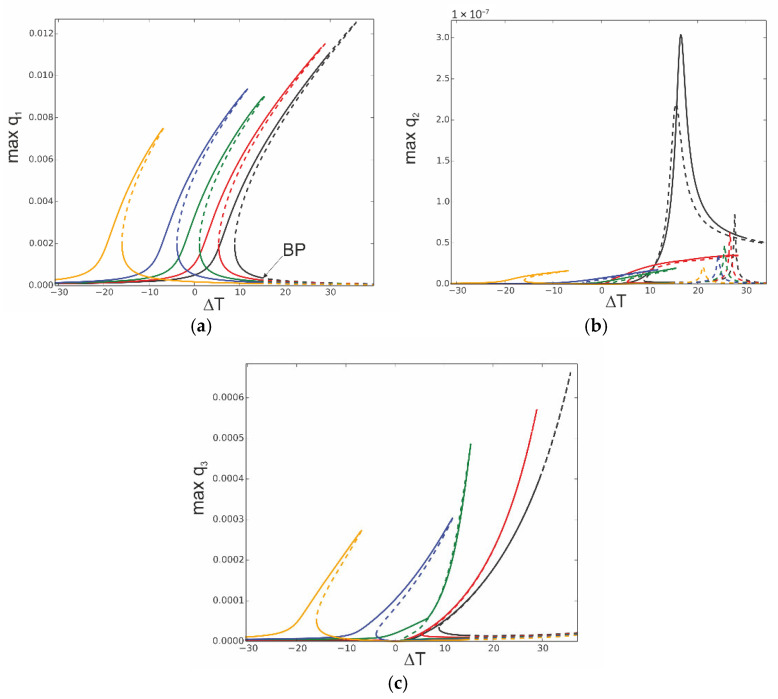
Bifurcation diagram of beam response against elevated temperature Δ*T* for fixed mechanical loading f_1_ = 0.1 × 10^−5^ and ω = 0.07; BP (bifurcation point) at Δ*T* = 14.35. (**a**) Coordinate q_1_, (**b**) coordinate q_2_, and (**c**) coordinate q_3_.

The bifurcation diagram of the beam response is obtained for a mechanical excitation level of f_1_ = 0.1 × 10^−5^ and for five different frequencies selected around the resonance zone. The shapes of the curves obtained against elevated temperature Δ*T* resemble the frequency response curves: ω = 0.05—black; ω = 0.06—red; ω = 0.07—green; ω = 0.08—blue; and ω = 0.10—orange (Figure 9a). It is interesting that for all curves the bifurcation point (BP) occurs for the same temperature value, about Δ*T* = 14.35. The involvement of the coordinate q_2_ (Figure 9b) is negligible, while q_3_ is one order smaller than q_1_ (Figure 9c).

**Figure 10:**
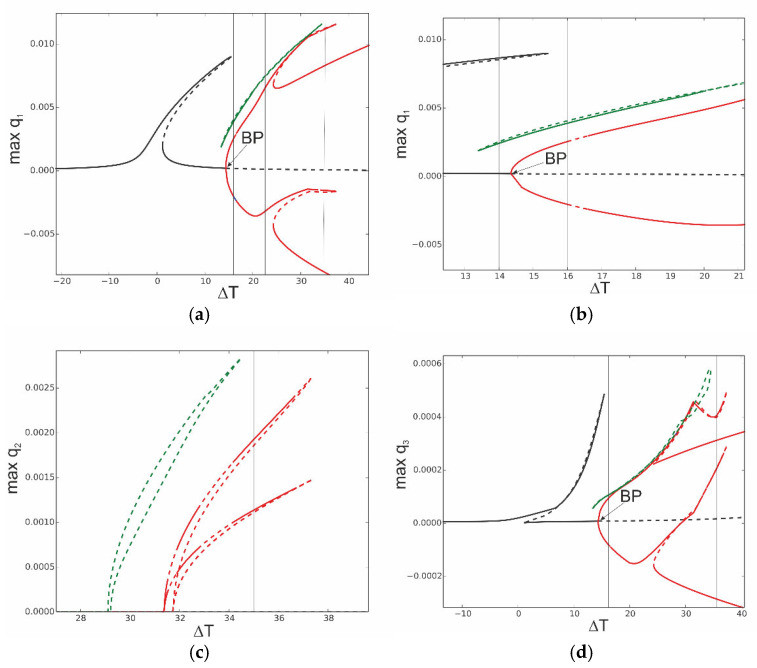
Bifurcation diagram of beam response against elevated temperature ∆*T* for fixed mechanical loading f_1_ = 0.1 × 10^−5^ and ω = 0.07; BP (bifurcation point) at ∆*T* = 14.35; post-buckling oscillations—red curves; isolated solution—green colour; (**a**) coordinate q_1_, (**b**) zoom of coordinate q_1_ in the vicinity of BP point, (**c**) coordinate q_2_, and (**d**) coordinate q**_3_**.

Detailed bifurcation analysis is conducted for fixed f_1_ = 0.1 × 10^−5^ and ω = 0.07 (Figure 10). Different branches of the solutions are indicated by dedicated colours. Starting from negative values of ∆*T*, we follow the black curve with the periodic response corresponding to excitation frequency. For negative temperature coordinates, q_1_, q_2_, and q_3_ become very small values (Figure 10c,d).

Approaching ∆*T* = 0 and going forward, the beam response increases up to the maximal value at the turning point ∆*T* = 15.43. The bifurcation curve resembles a stiffening resonance curve with three existing solutions.

Moving forward, the periodic solution bifurcates at point BP (∆*T* = 14.35), which is visible for the first and third vibration modes in Figure 10a,b,d. After buckling, two branches arise (red curves). They are asymmetric, which is more visible for larger values of ∆*T*. Around the elevated temperature ∆*T* = 32, the second mode is strongly involved in the beam dynamics, as presented in Figure 10c.

Apart from the periodic solutions obtained by the continuation method, the isolated solutions shown in Figure 10a by the green “isola” above the red branch are also obtained. The isolated solutions are partially stable and partially unstable (solid or dashed green line).

**Figure 11:**
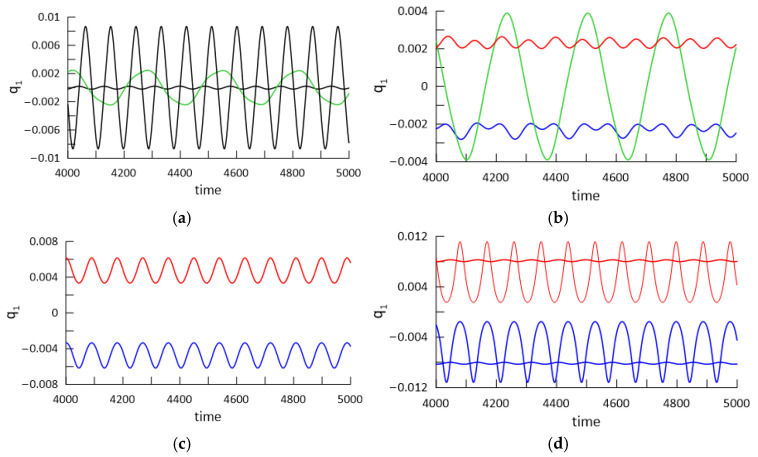
Time histories of beam response for selected values of elevated temperature ∆*T* and mechanical loading: f_1_ = 0.1 × 10^−5^ and ω = 0.07; (**a**) ∆*T* = 14, (**b**) ∆*T* = 16, (**c**) ∆*T* = 22, and (**d**) ∆*T* = 35.

To demonstrate beam dynamics and coexisting solutions, the time histories are plotted for the selected temperature values: ∆*T* = 14, ∆*T* = 16, ∆*T* = 22, and ∆*T =* 35. The values of selected ∆*T* are indicated by thin vertical lines on the bifurcation diagram in Figure 10. The time histories are shown in Figure 11a–d. For ∆*T =* 14 (Figure 11a), we obtain two periodic solutions corresponding to the upper and lower branches in the bifurcation diagram (black curve). However, there is a third stable solution (green line) with a period that is about tripled. This solution corresponds to the green “isola” on the bifurcation diagram. For ∆*T* = 16 (Figure 11b), after the bifurcation point, post-buckling oscillations of the beam with a positive (red) or negative (blue) offset take place. But again, large-amplitude oscillations (green colour) exist with a tripled period, and they correspond to oscillations between both buckled states. For the higher temperature ∆*T* = 22, just two small-amplitude vibrations around the buckled states exist, plotted by the red and blue time histories in Figure 11c. For this temperature, the solutions of the “isola” are unstable; therefore, the large-amplitude oscillations presented by a green line in Figure 11b do not exist anymore. For the higher temperature ∆*T* = 35, four different post-buckling vibrations are possible (Figure 11d). This is a result of the third and the second mode activation, which is visible in Figure 10c,d. It means that for ∆*T* = 35 we obtain large- and small-amplitude oscillations around the buckled states with a positive (red) and negative (blue) offset.

**Figure 12:**
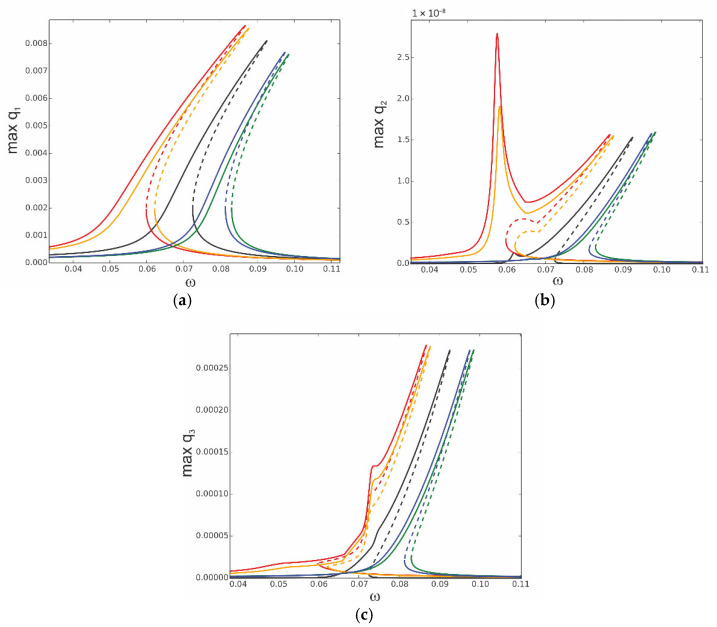
Resonance curves around the first natural frequency for amplitude of excitation f1 = 0.1 × 10^−5^ and different values of distributed temperature: *T*_up_ = 10, *T*_d_ = 0—red; *T*_up_ = 0, *T*_d_ = 10—orange; *T*_up_ = −10, *T*_d_ = 0—green; *T*_up_ = 0, *T*_d_ = −10—blue; reference curve: *T*_up_ = 0, *T*_d_ = 0—black; (**a**) coordinate q_1_, (**b**) coordinate q_2_, and (**c**) coordinate q_3_.

The proposed model enables the study of the effect of heat transfer linearly distributed through the beam thickness, assuming different temperatures of the upper (*T*_up_) and lower (*T*_d_) beam surfaces. The resonance curves for different temperature gradients are shown in Figure 12. The reference resonance curve for *T*_up_ = 0 and *T*_d_ = 0 is plotted in black. For the temperature gradient *T*_up_ = 10 and *T*_d_ = 0, the resonance curve (red colour) is shifted to lower frequencies; for the opposite temperature gradient, *T*_up_ = 0 and *T*_d_ = 10, we observe the same trend, but the shift is smaller (orange curve). This phenomenon comes from the asymmetric geometric and material properties of the beam. In the case of negative temperature, for *T*_up_ = −10 and *T*_d_ = 0, the resonance curve is shifted towards higher frequencies (green curve), and for *T*_up_ = 0 and *T*_d_ = −10, again, the shift is smaller (blue curve).

**Figure 13:**
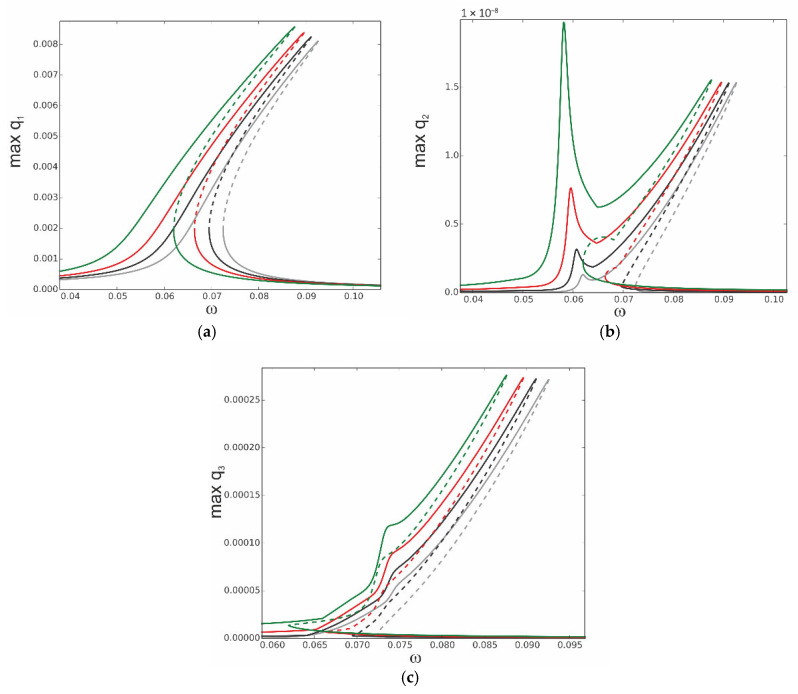
Resonance curves around the first natural frequency for mechanical loading f_1_ = 0.1 × 10^−5^ and different gradients of linear temperature distribution: *T*_up_ = 15, *T*_d_ = −15—black; *T*_up_ = −30, *T*_d_ = −30—red; *T*_up_ = 50, *T*_d_ = −50—green; reference resonance curve for *T*_up_ = 0, *T*_d_ = 0—grey; (**a**) coordinate q_1_, (**b**) coordinate q_2_, and (**c**) coordinate q_3_.

Similar analysis is repeated for a larger temperature gradient by assuming temperatures on the upper and bottom surfaces with opposite signs. The resonance curves in Figure 13 are computed for *T*_up_ = 15 and *T*_d_ = −15 (black), *T*_up_ = 30 and *T*_d_ = −30 (red), and *T*_up_ = 50 and *T*_d_ = −50 (green). Comparing with the reference curve *T*_up_ = 0 and *T*_d_ = 0 plotted in grey, in all cases, the curves are shifted towards lower frequencies when the temperature gradient is increased.

It should be noted that for the above studied temperature gradients the beam’s buckling does not occur. Even for the large temperature difference *T*_up_ = 50 and *T*_d_ = −50, the curve is just slightly more shifted. This is a result of two opposite effects, positive temperature on the upper part of the beam and negative on the lower part, which compensate for each other. For the elevated temperature, when the beam is uniformly heated, the buckling occurs at ∆*T* = 14.35.

**Figure 14:**
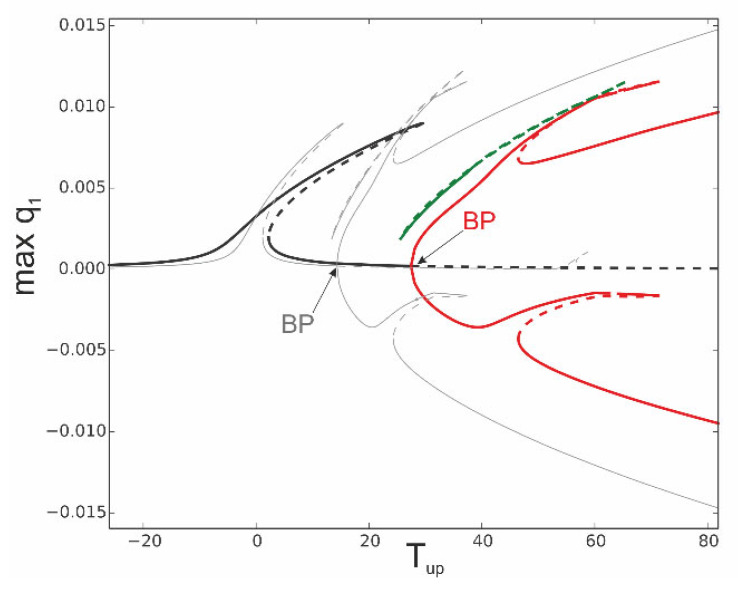
Bifurcation diagram of coordinate q_1_ against temperature of the upper beam surface *T*_up_ for fixed temperature of bottom surface *T*_d_ = 0: reference response against elevated beam temperature Δ*T*—grey curve; fixed mechanical loading, f_1_ = 0.1 × 10^−5^ and ω = 0.07.

To observe a difference in the bifurcation scenario, we select *T*_up_ as a bifurcation parameter while the temperature of the bottom surface is fixed, *T*_d_ = 0 (Figure 14). Then, the *T*_up_ temperature is varied from about *T*_up_ = −25 up to about *T*_up_ = 82. We can distinguish pre-buckling solutions (black), post-buckling (red), and isolated solutions (green). The bifurcation diagram against elevated temperature ∆*T* plotted in Figure 10 is now repeated as a reference in Figure 14 as a thin grey line. The bifurcation diagram against *T*_up_ shows a much stronger stiffening nature, and the bifurcation point of buckling takes a larger value *T*_up_ = 27.44 (red) compared to the BP indicated in grey. Furthermore, the post-bucking solutions and isolated green solutions are shifted towards higher temperatures. Nevertheless, the solutions for linearly distributed temperature demonstrate qualitative similarities with the elevated temperature of the beam.

**Figure 15:**
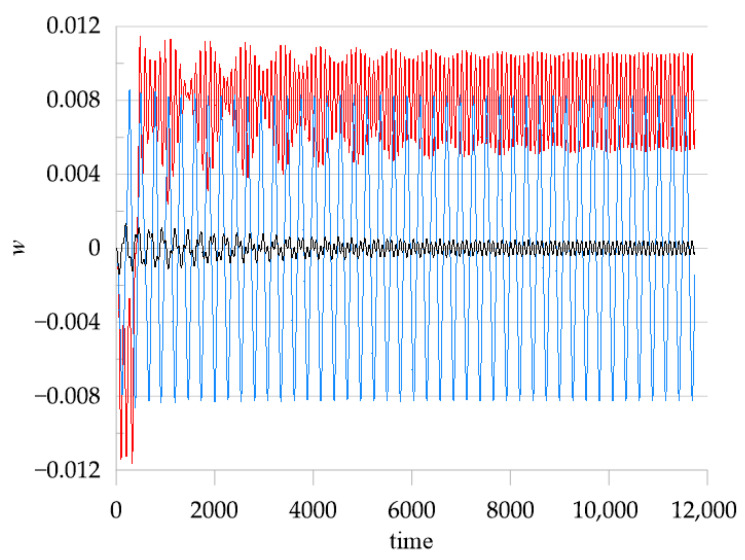
Time history diagrams of the response of the beam centre. Black line: *T*_up_ = 15, *T*_d_ = 10; blue line: *T*_up_ = 26, *T*_d_ = 10; red line: *T*_up_ = 35, *T*_d_ = 10. f_1_ = 0.1 × 10^−5^ and ω = 0.07.

In Figure 15, time history diagrams for three different cases of linear distribution of the temperature along the beam thickness are shown. In all cases, the temperature of the down surface of the beam is 10, and the excitation frequency is 0.07. For a small change in temperature when *T*_up_ = 15 (black curve), the amplitude of vibrations is small. For *T*_up_ = 26 (blue curve), the temperature difference leads to additional bending, and the amplitudes of vibrations become quite large. For a bigger temperature difference, when *T*_up_ = 35 (red curve), the beam buckles and continues to vibrate around the new equilibrium state.

**Figure 16:**
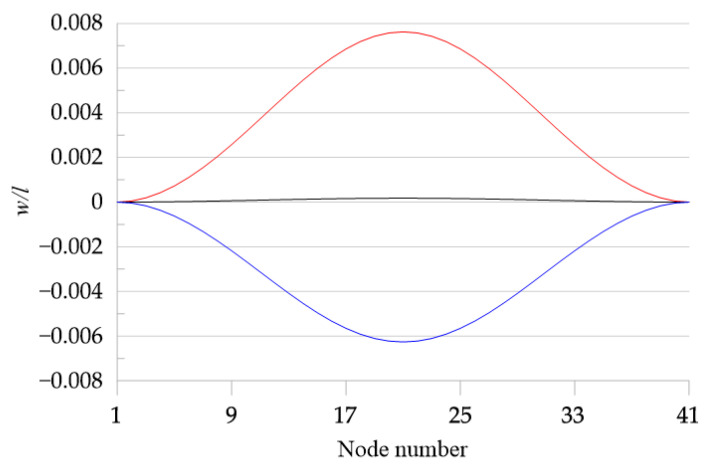
Displacement along the beam length for a fixed moment in time t = 6554. Black line: *T*_up_ = 15, *T*_d_ = 10; blue line: *T*_up_ = 26, *T*_d_ = 10; red line: *T*_up_ = 35, *T*_d_ = 10. f_1_ = 0.1 × 10^−5^ and ω = 0.07.

The deflections along the beam length at a selected time (time = 6554) are shown in Figure 16. Here, the big difference in the amplitudes of the beam with just small temperature differences and the ones with bigger differences is clearly visible.

**Figure 17:**
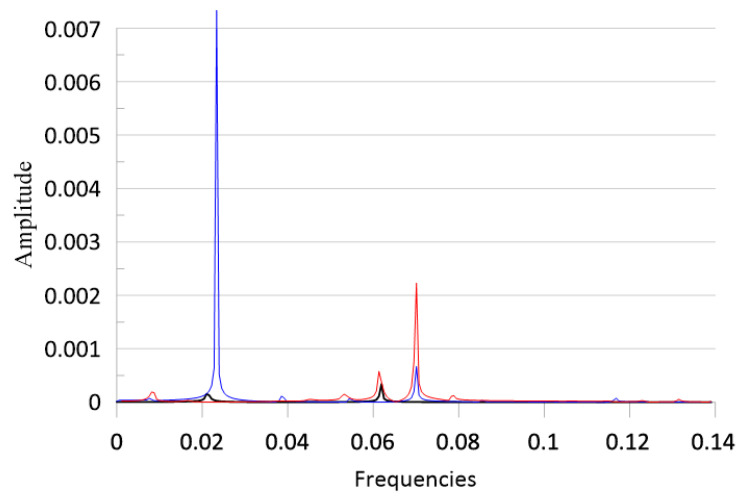
FFT of the responses of the time series plotted in Figure 15. *T*_up_ = 15, *T*_d_ = 10; blue line: *T*_up_ = 26, *T*_d_ = 10; red line: *T*_up_ = 35, *T*_d_ = 10. f_1_ = 0.1 × 10^−5^ and ω = 0.07.

The results of the fast Fourier transforms of the time series plotted in Figure 15 are shown in Figure 17, maintaining the same colour set. The influence of the first natural frequency on the response is clearly seen in all three cases. The excitation frequency ω = 0.07 is also clearly visible in the figure. It is an interesting fact that in the case of *T*_up_ = 26, for ω = 0.023, a large peak in the spectra is seen with a frequency about three times smaller than the excitation frequency. This oscillation represents beam motion between two buckled states. Close to this value of frequency, there is a small peak in the curve obtained for *T*_up_ = 15. For *T*_up_ = 35, some small additional peaks appear for lower and higher frequencies, demonstrating nonlinear dynamics and the possible existence of secondary sub- or super- harmonic resonances.

## 7. Applicability of One-Mode Reduction

As mentioned above, the use of the one-mode reduction model would provide significant advantages in studying the nonlinear dynamics of a beam, if this model can be validated. The examples below show the applicability of this model as well as its limitations.

**Figure 18:**
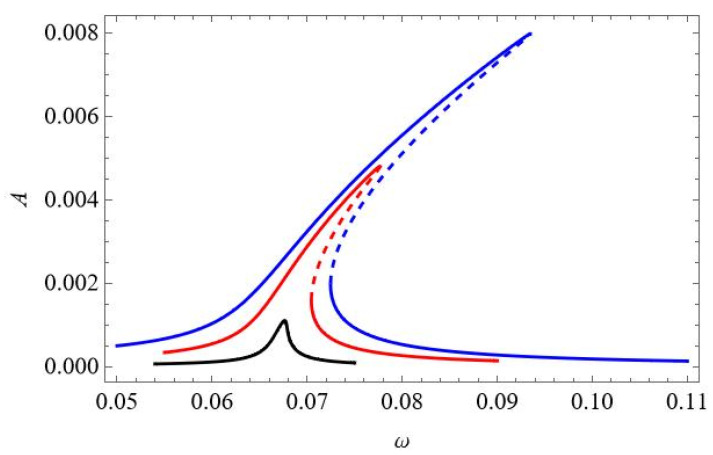
Resonance curves around the first natural frequency for small mechanical loading: f1 = 0.1 × 10^−6^—black; f1 = 0.5 × 10^−6^—red; f1 = 0.1 × 10^−5^—blue; ∆*T* = 0.

In Figure 18, the results obtained by the 1-DoF model, using the same data as those used to obtain Figure 4a by the 3-DoFs model, are shown. As one can see, the results are in perfect agreement. Obviously, for these low levels of load amplitudes and ∆*T* = 0, the 1-DoF model is enough to obtain correct results.

**Figure 19:**
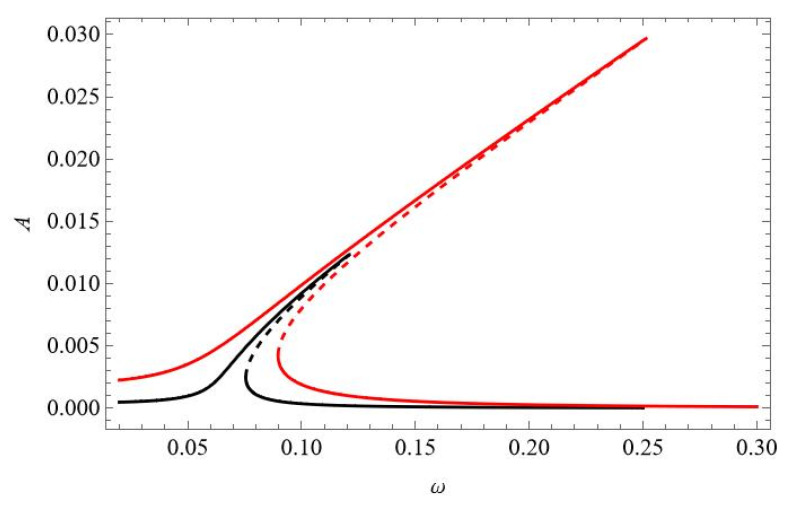
Resonance curves around the first natural frequency for large mechanical loading: f_1_ = 0.2 × 10^−5^—black; f_1_ = 0.1 × 10^−4^—red; f_2_ = 0; f_3_ = 0; ∆*T* = 0.

The results shown in Figure 19 correspond to the case presented in Figure 5. The amplitude of the loading is much bigger than in the previous case. This leads to essential changes in the resonance curves obtained by the different models. The amplitudes of vibrations in the case of the 1-DoF model are larger, but more significant is the fact that the 3-DoFs model predicts instability of the upper branch of the resonance curve. This cannot be obtained by a 1-DoF model.

**Figure 20:**
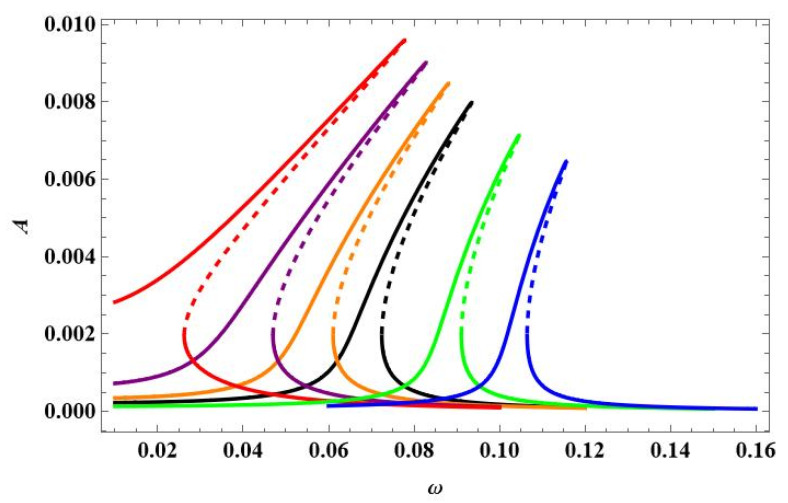
Resonance curves around the first natural frequency for mechanical loading f_1_ = 0.1 × 10^−5^ and different values of elevated temperature: ∆*T* = 0—black; ∆*T* = 5—orange; ∆*T* = 10—magenta; ∆*T* = 15—red; ∆*T* = −10—green; ∆*T* = −20—blue.

Most of the curves shown in Figure 20, plotted for different temperatures, are very similar to those shown in Figure 7a. However, for ∆*T* = 15, the additional instability obtained by the 3-DoFs model cannot be predicted. Of course, the interaction of modes and partially stable and partially unstable isolated solutions are obtained only by the 3-DoFs model for higher temperatures, and the 1-DoF model cannot obtain them.

**Figure 21:**
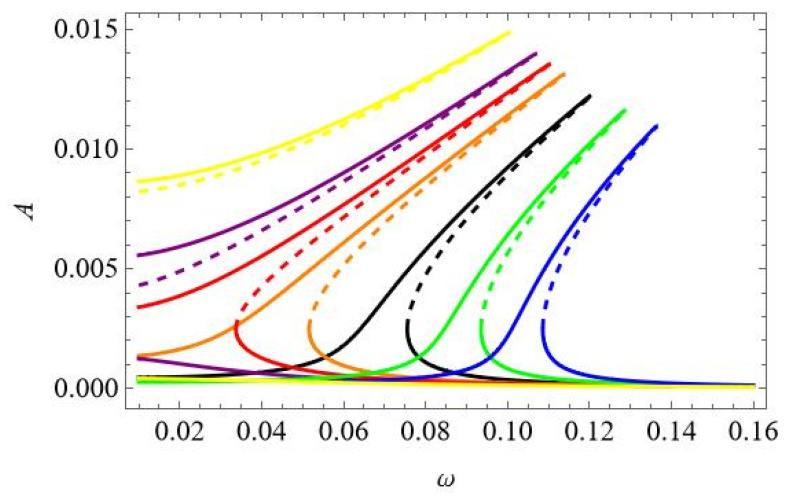
Resonance curves around the first natural frequency for mechanical loading f_1_ = 0.2 × 10^−5^ and different values of elevated temperature: ∆*T* = 0—black; ∆*T* = 10—orange; ∆*T* = 15—red; ∆*T* = 20—magenta; ∆*T* = 30—yellow; ∆*T* = −10—green; ∆*T* = −20—blue.

If one compares Figure 21 with Figure 8, the same conclusion can be made—multiple solutions (Δ*T* = 15) and many instabilities in the solutions cannot be detected by the 1-DoF model.

**Figure 22:**
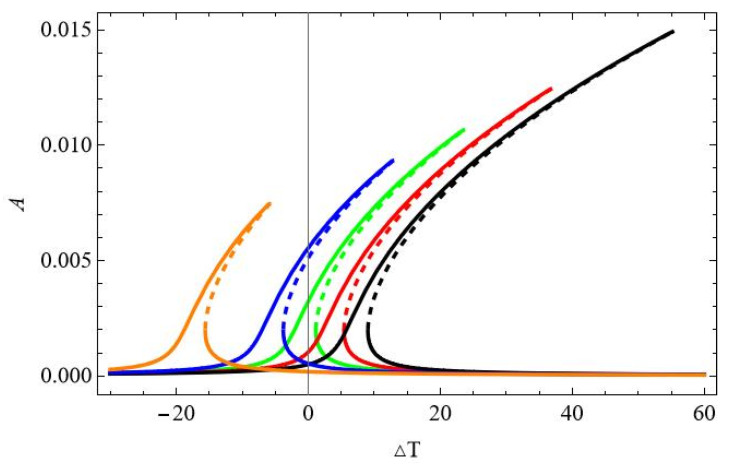
Bifurcation diagram of the beam response against elevated temperature ∆*T* in the vicinity of the first natural frequency and fixed mechanical loading f_1_ = 0.1 × 10^−5^ and different excitation frequencies: ω = 0.05—black; ω = 0.06—red; ω = 0.07—green; ω = 0.08—blue; ω = 0.10—orange.

The bifurcation diagrams in Figure 22 show similar behaviour of the beam response q_1_ as the one in Figure 9a, but the values of the maximal amplitudes are different, and some instability in the upper branches of the curve for the maximal temperature is not detected by the 1-DoF model.

In the case of a linear distribution of the temperature along the beam thickness, the results obtained analytically are similar to those shown in Figure 12a. The asymmetry due to the geometric and material properties of the layers is also confirmed analytically.

## 8. Conclusions

In this study, a nonlinear thermoelastic model of a bimaterial beam is developed, considering mechanical and thermal boundary conditions. The model enables the study of the influence of mechanical and thermal loading, considering the heat linearly distributed through the beam thickness. The model is validated through comparison with finite element analysis, including vibration modes, frequencies, and time response under periodic excitation for clamped–clamped boundary conditions.

The impact of mechanical and thermal loading is investigated for the three-degrees-of-freedom model and then compared with the analytical solutions obtained by the harmonic balance method from the reduced one-degree-of-freedom model. The models with one and three degrees of freedom exhibited strong agreement for relatively small or moderate periodic forces, demonstrating that higher vibration modes (second and third) have a minimal effect on the system’s behaviour. The resonance curves showed stiffening, particularly at larger amplitudes. However, for large-amplitude oscillations of the bimaterial beam, an instability zone occurs in the frequency response curve because of a strong interaction of the second and third vibration modes, resulting in additional solutions. The reduced model based on one mode cannot detect this phenomenon. Moreover, the increased temperature leads to bifurcation and bimaterial beam buckling with asymmetric post-buckling oscillations and strong modal interactions. Then, even four stable solutions may exist with small- or large-amplitude oscillations around each of the two buckled states, and also, the isolated solution of a large amplitude with oscillations between two buckled states may occur. The shift of the resonance curves and a nonlinear stiffening effect is observed in the case of linearly distributed temperature throughout the beam thickness. However, if the sign of the temperature gradient is changed, the shift of the curve is different. By increasing the temperature of the upper beam surface and fixing the temperature of the bottom, a stronger hardening phenomenon is obtained compared with the case of elevated temperature. The bifurcation point occurs at a higher temperature, and thus, post-buckling dynamics are shifted as well. However, the beam response is in qualitative agreement with the response for the beam under elevated temperature.

In conclusion, the analysis underscores the importance of considering mechanical and thermal effects, particularly at elevated temperatures, to accurately predict the dynamic behaviour of bimaterial beams under periodic loading. The findings highlight the significance of higher vibration modes, temperature distribution, and post-buckling dynamics, contributing valuable insights into the design and performance of such systems.

The obtained results demonstrate the possibility of transitioning to more complex chaotic oscillations, and this problem will be investigated by a detailed analysis of bifurcation diagrams, basins of attractions, Poincaré maps, Lyapunov exponents, and Fourier transformation in future studies, considering the coupling of mechanical and thermal fields.

## Figures and Tables

**Table 1:** Natural frequencies obtained with different meshes.

Number	Mesh with 6000 Elements, Hz	Mesh with 32,000 Elements, Hz	Mesh with 256,000 Elements, Hz
1	121.78	117.39	117.22
2	335.40	323.30	322.87
3	656.84	633.20	632.38

**Table 2:** The natural frequencies of the beam according to the finite element model and the Timoshenko beam theory for the homogenized beam.

No	Reduced Model Hz	Finite Element Model Hz	Difference %
1	108.77	117.39	7.3
2	299.53	323.30	7.35
3	586.42	633.20	7.38

## Data Availability

The original contributions presented in this study are included in the article. Further inquiries can be directed to the corresponding author.

## Nomenclature

*x*	Longitudinal coordinate
*z*	Transverse coordinate
*u*	Displacement in longitudinal direction
*w*	Displacement in transverse direction
*t*	Time
*l*	Length of the beam
*b*	Width of the beam
z_m_	The coordinate of interface layer
$h^{\left(i\right)}$	Thickness of *i*th layer
*h* = *h*^(1)^ + *h*^(2)^	Thickness of the beam
$\psi \left(x,t\right)$	Angle of rotation of the normal to the mid-surface of the beam
ε	Strain vector
$\varepsilon_{x}^{0}$ $\varepsilon_{xz}^{0}$	Components of strain in the mid-axes
$\kappa_{x}^{0}$	Curvature in the mid-axes
$k^{2}$	Shear correction factor, depending on the geometry; for a rectangular section, taken as $k^{2}$ = 5/6
$S=\left\{\sigma_{x},\sigma_{xz}\right\}$	Stress vector
$\sigma_{x}^{\left(i\right)}$	Normal stress in *i*th layer
$\sigma_{xz}^{\left(i\right)}$	Tangential stress in *i*th layer
$E^{\left(i\right)}$	Young’s modulus of *i*th layer
$G^{\left(i\right)}$	Shear modulus of *i*th layer
$\alpha_{T}^{\left(i\right)}$	Coefficient of linear temperature expansion of *i*th layer
$\bar{Eh}=E^{\left(1\right)}h^{\left(1\right)}+E^{\left(2\right)}h^{\left(2\right)}$ $\bar{Gh}=G^{\left(1\right)}h^{\left(1\right)}+G^{\left(2\right)}h^{\left(2\right)}$ $\bar{E}\bar{I}=\frac{1}{3}\left\{E^{\left(1\right)}\left(z_{1}^{3}+\frac{h^{3}}{8}\right)+E^{\left(2\right)}\left(\frac{h^{3}}{8}-z_{1}^{3}\right)\right\}$	Generalized flexural beam’s rigidity
T	Difference between the temperature of the beam and the ambient temperature
T_d_, T_up_	Temperature on bottom (down) and upper beam surfaces
p(x,t)	Transverse loading
*c*_1_, *c*_2_	Damping coefficients
*d*_1_, *d*_2_	Dimensionless damping coefficients
$W_{i}(x)$	Normal-mode shape of beam transverse displacement
$\Psi_{i}(x)$	Normal-mode shape of beam rotation angle
*q*_i_(t)	Generalized coordinate, time function
ω_n_	Natural frequencies of the linear elastic undamped beam
P, ω	Amplitude and frequency of harmonic excitation
*ξ*_n_	Modal damping coefficient

## Appendix A

The coefficients in Equation (34) are obtained by using Equations (27), (31), (33), and (35). For the particular case considered in the paper, they have the following numerical values:

$C_{(1)111}$ = 0.81681 × 10^2^	$C_{(1)222}$ = −0.62987 × 10^−2^	$C_{(1)333}$ = −0.51910 × 10^3^
C_(1)112_ = −0.50505 × 10^−2^	C_(1)113_ = −0.19388 × 10^3^	C_(1)122_ = 0.30558 × 10^3^
C_(1)223_ = −0.24185 × 10^3^	C_(1)133_ = 0.75817 × 10^3^	C_(1)233_ = −0.13175 × 10^−1^
C_(1)123_ = 0.48940 × 10^−2^		
C_(2)111_ = −0.16836 × 10^−2^	C_(2)222_ = 0.11432 × 10^4^	C_(2)333_ = −0.17481 × 10^−2^
C_(2)112_ = 0.30556 × 10^3^	C_(2)113_ = 0.24461 × 10^−2^	C_(2)122_ = −0.18894 × 10^−1^
C_(2)223_ = −0.24399 × 10^−2^	C_(2)133_ = −0.13175 × 10^−1^	C_(2)233_ = 0.24536 × 10^4^
C_(2)123_ = −0.48346 × 10^3^		
C_(3)111_ = −0.64642 × 10^2^	C_(3)222_ = −0.81442 × 10^−3^	C_(3)333_ = 0.52654 × 10^4^
C_(3)112_ = 0.24469 × 10^−2^	C_(3)113_ = 0.75800 × 10^3^	C_(3)122_ = −0.24184 × 10^3^
C_(3)223_ = 0.24532 × 10^4^	C_(3)133_ = −0.15566 × 10^4^	C_(3)233_ = −0.52363 × 10^−2^
C_(3)123_ = −0.26341 × 10^−1^		
D_111_ = −0.28030 × 10^−3^	D_112_ = 0.57776 × 10^−8^	D_113_ = 0.22185 × 10^−3^
D_121_ = 0.57775 × 10^−8^	D_122_ = −0.10486 × 10^−2^	D_123_ = 0.74706 × 10^−9^
D_131_ = 0.22183 × 10^−3^	D_312_ = 0.74705 × 10^−9^	D_133_ = −0.22502 × 10^−2^
D_211_ = 0.51769 × 10^−7^	D_212_ = −0.10670 × 10^−11^	D_213_ = 0.40972 × 10^−7^
D_221_ = 0.10670 × 10^−11^	D_222_ = −0.19366 × 10^−6^	D_223_ = 0.13797 × 10^−12^
D_231_ = 0.40969 × 10^−7^	D_232_ = 0.13797 × 10^−12^	D_233_ = −0.41559 × 10^−6^
ω_1_ = 0.63799 × 10^−1^	ω_2_ = 0.17570	ω_3_ = 0.34402
